# Evaluation of isthmin-1 immunoreactivity in gastric adenocarcinoma

**DOI:** 10.1590/0102-672020260000013e1942

**Published:** 2026-08-07

**Authors:** Onur AĞ, Mesut YUR, Serhat HANÇER, Mehmet Bugra BOZAN, Ibrahim Hanifi ÖZERCAN, Tuncay KULOGLU

**Affiliations:** 1 University of Health Sciences, Elazig Fethi Sekin City Hospital, Department of General Surgery - Elazig, Turkey.; 2 Firat University, Faculty of Medicine, Department of General Surgery - Elazig, Turkey.; 3 Firat University, Faculty of Medicine, Department of Histology and Embryology - Elazig, Turkey.; 4 Firat University, Faculty of Medicine, Department of Medical Pathology - Elazig, Turkey.

**Keywords:** Adenocarcinoma, Stomach neoplasms, Metaplasia, Stomach diseases, Adipokines, Adenocarcinoma, Neoplasias gástricas, Metaplasia, Gastropatias, Adipocinas

## Abstract

**Background:**

Isthmin-1, a recently discovered adipokine, was first characterized for its role in early brain development. Subsequent studies have demonstrated that isthmin-1 is involved in a broad range of biological processes, including metabolism, immunity, tumorigenesis, cellular proliferation, endothelial permeability, and organ development.

**Aims:**

This study aimed to evaluate isthmin-1 immunoreactivity in tumor tissues obtained from gastric cancer patients using immunohistochemistry and to assess its usefulness in the diagnosis and histological evaluation of gastric cancer.

**Methods:**

The patients were divided into three groups: adenocarcinoma, intestinal metaplasia, and healthy controls. Endoscopic biopsies, obtained according to the Sydney protocol, were examined for the control group and patients diagnosed with intestinal metaplasia. In contrast, surgical resection tissues were used for patients diagnosed with gastric adenocarcinoma.

**Results:**

All materials were examined histopathologically. No significant difference in isthmin-1 levels was detected between the control and intestinal metaplasia groups, based on immunohistochemical staining. However, when gastric adenocarcinoma cases were compared with the control and intestinal metaplasia groups, a significant decrease in isthmin-1 immunoreactivity was observed in adenocarcinoma cases. Patients with adenocarcinoma were grouped as well-differentiated, moderately differentiated, and poorly differentiated, and then staged according to the TNM staging system. Histopathological examination revealed no significant differences in the degree of differentiation or TNM stage.

**Conclusions:**

We believe that isthmin-1 can be used as a biomarker in the diagnosis of malignant gastric diseases in the future. However, more comprehensive and extensive scientific studies are needed to determine its usefulness in tumor staging or prognosis monitoring.

## ARTICLE HIGHLIGHTS

• Isthmin-1 can be used as a biomarker in the diagnosis of malignant gastric diseases in the future.

• Isthmin-1 plays an active role in tumorigenesis.

• Adipokines may play an even more important role in the diagnosis and prognosis of gastric cancer in the future.

• With the increasing life expectancy after tumor surgery, the importance of these markers will also increase.

## Summary

**CENTRAL MESSAGE:**

The development of gastric cancer involves a complex interplay of genetic predispositions and environmental exposures, progressing from normal epithelium to invasive cancer and metastasis. Advances in molecular oncology, including cancer genetic profiling and the identification of tumor biomarkers, have paved the way for personalized therapeutic strategies. Adipokines - bioactive peptides secreted by adipose tissue - are involved in various physiological and pathological processes, including immune modulation, lipid metabolism, inflammation, vascular regulation, and oxidative stress. isthmin-1 (ISM1) is a recently discovered adipokine that has been shown to be involved in a broad range of biological processes, including immunity and tumorigenesis.

**PERSPECTIVES:**

When adenocarcinoma cases were compared with the control and intestinal metaplasia groups, a significant decrease in isthmin-1 immunoreactivity was observed in adenocarcinoma cases. No significant difference was found between the staging and differentiation of adenocarcinoma cases, which were classified into three groups: well-differentiated, moderately differentiated, and poorly differentiated according to TNM staging. Based on these results, we believe that isthmin-1 could be used as a biomarker in the diagnosis of gastric carcinoma in the future.

## VISUAL ABSTRACT

**Figure 1:**
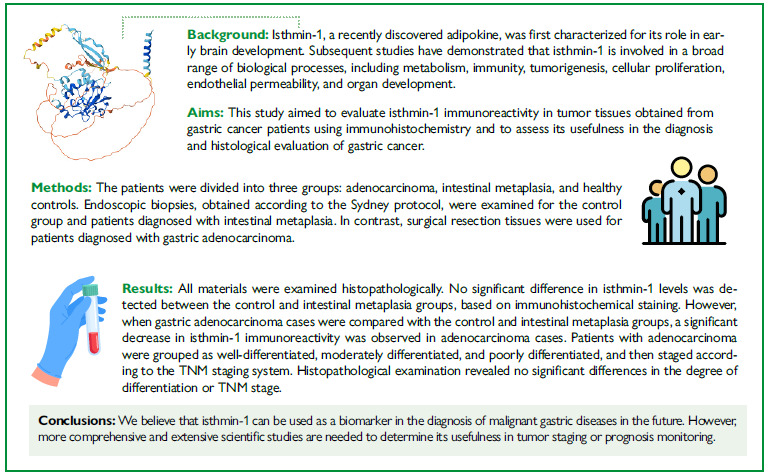

## RESUMO

**Racional:**

A istmina-1, uma adipocina descoberta recentemente, foi primeiro caracterizada por seu papel no desenvolvimento cerebral inicial. Estudos subsequentes demonstraram que a istmina-1 está envolvida em uma ampla gama de processos biológicos, incluindo metabolismo, imunidade, tumorigênese, proliferação celular, permeabilidade endotelial e desenvolvimento de órgãos.

**Objetivos:**

Este estudo teve como objetivo avaliar a imunorreatividade da istmina-1 em tecidos tumorais obtidos de pacientes com câncer gástrico por meio de imunohistoquímica e avaliar sua utilidade no diagnóstico e avaliação histológica do câncer gástrico.

**Métodos:**

Os pacientes foram divididos em três grupos: adenocarcinoma, metaplasia intestinal e controles saudáveis. Biópsias endoscópicas, obtidas de acordo com o protocolo de Sydney, foram examinadas para o grupo controle e para os pacientes diagnosticados com metaplasia intestinal. Em contrapartida, tecidos obtidos de ressecção cirúrgica foram utilizados para os pacientes diagnosticados com adenocarcinoma gástrico.

**Resultados:**

Todos os materiais foram examinados histopatologicamente. Nenhuma diferença significativa nos níveis de istmina-1 foi detectada entre os grupos controle e de metaplasia intestinal com base na análise imunohistoquímica. Entretanto, quando os casos de adenocarcinoma gástrico foram comparados com os grupos controle e de metaplasia intestinal, observou-se uma diminuição significativa na imunorreatividade da istmina-1 nos casos de adenocarcinomas. Os portadores de adenocarcinoma foram agrupados como bem diferenciados, moderadamente diferenciados e pouco diferenciados, e então estadiados de acordo com o sistema de estadiamento TNM. O exame histopatológico não revelou diferenças significativas no grau de diferenciação ou no estadiamento TNM.

**Conclusões:**

Acreditamos que a istmina-1 pode ser usada como um biomarcador no diagnóstico de doenças gástricas malignas no futuro. No entanto, estudos científicos mais abrangentes e extensos são necessários para determinar sua utilidade no estadiamento tumoral ou no monitoramento do prognóstico.

## INTRODUCTION

Gastric cancer is a prevalent malignancy worldwide, with approximately one million new cases and 650,000 deaths reported annually, placing it among the leading causes of cancer-related mortality globally^21^. Its incidence is nearly twice as high in men as in women^18^. Due to its biological heterogeneity and aggressive clinical progression, gastric cancer remains a substantial public health concern across the world^7^.

Stomach cancer is generally diagnosed at an advanced stage in developed countries due to the lack of specific early symptoms and the relatively low incidence in these regions^15^. While overall incidence rates have declined in recent years, delays in medical treatment persist.

The development of gastric cancer involves a complex interplay of genetic predispositions and environmental exposures, progressing from normal epithelium to invasive cancer and metastasis. Key established risk factors include *Helicobacter pylori* infection, male sex, family history of gastric cancer, and tobacco use. Dietary habits, such as frequent intake of salted foods, high-nitrite processed products, and insufficient consumption of fruits and vegetables, also contribute significantly to disease risk^3^. In addition, mutations in genes such as *Cadherin 1* (CDH1) and *Adenomatous Polyposis Coli* (APC) have been implicated in the molecular pathogenesis of gastric cancer^2^ ^,^ ^8^.

Advances in molecular oncology, including cancer genetic profiling and the identification of tumor biomarkers, have paved the way for personalized therapeutic strategies. Targeted treatments, including monoclonal antibodies and small-molecule inhibitors, have become integral components of multimodal gastric cancer therapy. A notable example is the HER2, a member of the human epidermal growth receptor 2 (EGFR) family, which plays a critical role in certain breast cancers and is reported to be overexpressed in approximately 15-37% of gastric cancers^9^.

Adipokines - bioactive peptides secreted by adipose tissue - are involved in various physiological and pathological processes, including immune modulation, lipid metabolism, inflammation, vascular regulation, and oxidative stress^6^. While they exert local effects via autocrine and paracrine signaling, adipokines also influence the function and growth of distant organs such as the heart, liver, pancreas, and brain^1^.

Isthmin-1 (ISM1), a recently discovered adipokine, was first characterized for its role in early brain development. Subsequent studies have demonstrated that ISM1 is involved in a broad range of biological processes, including metabolism, immunity, tumorigenesis, cellular proliferation, endothelial permeability, and organ development. It is most abundantly expressed in the placenta^6^. Additionally, ISM1 is secreted by mature brown and white adipocytes and has been shown to regulate glucose uptake, improve insulin sensitivity, and suppress hepatic lipid accumulation, underscoring its importance in metabolic homeostasis^1^ ^,^ ^11^.

Recent evidence suggests that ISM1 may also contribute to tumor biology by promoting cancer cell migration and dissemination in malignancies such as melanoma, hepatocellular carcinoma, and colorectal, gastric, and breast cancers^20^.

The aim of the present study was to evaluate ISM1 immunoreactivity in gastric cancer tissues using immunohistochemical methods and to explore its potential role in the diagnosis and histopathological assessment of gastric cancer.

## METHODS

### Ethical approval

Before starting this study, approval was obtained from the Fırat University Non-Interventional Research Ethics Committee with the decision dated 10.08.2023 and No. 2023/11-10. The study population consisted of patients diagnosed with gastric cancer whose pathology samples were processed at the Fırat University Hospital Pathology Department from January 2015 to January 2023. All procedures involving human data were conducted in accordance with institutional ethical guidelines and with the Declaration of Helsinki and its annexes. Given the retrospective character of the study, there was no need for informed consent.

### Patient selection

#### Study population and grouping

The participants were retrospectively categorized into three groups:

• Group 1 (Control): 20 individuals with normal gastric mucosa and no pathological findings confirmed by endoscopic biopsy performed according to the Sydney protocol.
• Group 2: 20 patients diagnosed with intestinal metaplasia based on endoscopic biopsy results performed according to the Sydney protocol.
• Group 3: 60 patients with histologically verified gastric adenocarcinoma identified through examination of surgical resection specimens.

Demographic and clinical parameters, including sex, age, height, weight, and body mass index (BMI), were recorded for each participant. Additionally, tumor-node-metastasis (TNM) classification, lymph node involvement, presence of distant metastasis, serum lipid levels (HDL, LDL, triglycerides), and tissue ISM1 expression levels were documented. All data were collected retrospectively from the hospital’s digital medical archive system.

### Inclusion criteria

• Patients aged over 18 years;
• Patients who underwent surgery for gastric adenocarcinoma from January 2015 to January 2023;
• Patients diagnosed with intestinal metaplasia through endoscopic biopsy;
• Controls whose biopsies were negative for both intestinal metaplasia and malignancy;
• Patients with BMI:
  ◦ Normal (18.9-24.9 kg/m^2^);
  ◦ Overweight (25.0-29.9 kg/m^2^);
  ◦ Type-I obesity (30.0-34.9 kg/m^2^);
  ◦ Type II obesity (35.0-39.9 kg/m^2^).

### Exclusion criteria

• Patients under 18 years of age;
• Patients with end-stage renal disease;
• Individuals with active infections or chronic inflammatory conditions;
• Individuals receiving immunosuppressive therapy;
• Patients with a history of hematologic disorders;
• Patients with severe obesity (BMI>40 kg/m^2^);
• Patients diagnosed with additional malignancies;
• Cases with incomplete medical records;
• Atrophic gastritis.

### Immunohistochemical analysis

Tissue samples, cut into 4-6 µm sections from paraffin-embedded blocks, were placed on slides coated with poly-L-lysine. After deparaffinization and alcohol gradient dehydration, antigen retrieval was performed using citrate buffer (pH 6.0) heated in a microwave oven at 750 W for 20 minutes. Slides were cooled at room temperature for 20 minutes and then washed three times in phosphate-buffered saline (PBS).

Endogenous peroxidase activity was blocked using a hydrogen peroxide solution for 5 minutes, followed by PBS rinsing. To minimize non-specific background staining, slides were incubated with Ultra V Block solution for 5 minutes.

Next, the tissue was treated with rabbit polyclonal anti-ISM1 antibody (dilution 1:200, Elabscience, E-AB-18133) and incubated for 60 minutes at room temperature in a humid chamber. After PBS washing, the sections were incubated with a biotinylated secondary antibody (anti-mouse/rabbit immunoglobulin G [IgG]) for 30 minutes, followed by treatment with streptavidin-peroxidase for an additional 30 minutes.

Staining was visualized using a chromogenic 3-Amino-9-ethylcarbazole (AEC) substrate solution and the slides were rinsed in phosphate-buffered saline (PBS). Counterstaining was carried out with Mayer’s hematoxylin, followed by distilled water rinses. Slides were mounted with high-volume mounting medium and evaluated under a Leica DM500 microscope with a Leica DFC295 camera.

Immunoreactivity was assessed using a histoscore system, calculated by multiplying the proportion of stained cells (scored as: 0.1 for <25%, 0.4 for 26-50%, 0.6 for 51-75%, 0.9 for 76-100%) by staining intensity (scored as: 0=none; 0.5=very weak; 1=weak; 2=moderate; 3=strong).

Histopathological classification and tumor grading were performed in accordance with the 2016 World Health Organization criteria^12^.

### Statistical analysis

Data analysis was performed using Statistical Package for the Social Sciences (SPSS), version 21 (International Business Machines Corporation). The Kolmogorov-Smirnov test was applied to determine the normality of the distribution. Based on the data characteristics, comparisons between groups were conducted using either one-way analysis of variance (ANOVA) or the Kruskal-Wallis test. Correlation coefficients were calculated using Pearson or Spearman tests, depending on data distribution.

Significantly different parameters were further evaluated through receiver operating characteristic (ROC) analysis to determine diagnostic sensitivity and specificity. For normally distributed parametric data results were given as mean±standard deviation (minimum-maximum values). For non-normally distributed parametric data, results were given as median (%95 confidence interval). Categorical variables were analyzed using the ꭓ^2^ test and presented as counts (n) and percentages (%). Survival outcomes were assessed through univariate and multivariate Cox regression analyses. A p-value of less than 0.05 was considered statistically significant.

## RESULTS

One hundred patients meeting the inclusion criteria were enrolled: 60 with gastric cancer, 20 with intestinal metaplasia, and 20 as controls. Of these, 59 (59%) were men and 41 (41%) were women. The mean age was 61 years (range: 36-90 years) (Tables 1 and 2).

**Table 1.** Demographic and laboratory characteristics of all patients.

Variable	
Age (years) (mean±SD)	60.85±15.82 (36-90)
Sex	
Male	59 (59%)
Female	41 (41%)
Total	100 (100%)
HDL (mg/dl)	38 (10-89)
LDL (mg/dl)	76.1 (10.1-198.0)
TG (mg/dl)	118 (44-430)
BMI (kg/m^2^)	24.19 (17-40)

SD: standard deviation; HDL: high-density lipoprotein; LDL: low-density lipoprotein; TG: triglycerides; BMI: body mass index.

**Table 2.** Comparison of demographic and laboratory findings between the groups.

Variable	Intestinal Metaplasia n=20	Control Group n=20	Gastric Cancer Group n=60	p-value
Age (years) (mean±SD)	56.9±18.7	54.9±14.9	64.15±14.4	0.034*
Sex (%)				
Male	8 (40)	12 (60)	39 (65)	0.140
Female	12 (60)	8 (40)	31 (35)	
HDL (mg/dl)	33 (20-56)	36 (21-89)	40 (10-76)	0.091
LDL (mg/dl)	59.5 (29-154)	90.5 (17-198)	76 (10.1-100.7)	0.119
TG (mg/dl)	99 (44-430)	120 (56-286)	123 (46-422)	0.434
BMI (kg/m^2^)	23.8 (18.9-40)	24.4 (20-29.4)	24.2 (17-30.5)	0.617

SD: standard deviation; HDL: high-density lipoprotein; LDL: low-density lipoprotein; TG: triglycerides; BMI: body mass index.

*p<0.05 is statistically significant.

Additionally, a multivariate analysis of the predictive factors was conducted, resulting in the additional table that follows (Table 3).

When the patients in the cancer group were examined according to TNM staging, no statistically significant difference was observed in the T and N stages (p=0.718) (Table 4).

**Table 3.** Multivariate analysis results of predictive factors for malignancy.

Dependent variable	B score	OR	F score	Sig.	95%CI		Partial η²*
					Lower bound	Upper bound	
Age	64.317	0.090	5.886	<0.001	60.097	68.536	0.108
HDL	45.195	0.099	6.441	<0.001	42.251	48.139	0.117
LDL	108.436	0.133	8.612	<0.001	99.565	117.307	0.151
TG	138.317	0.024	2.230	<0.001	118.870	157.764	0.044
BMI	23.867	0.043	3.206	<0.001	22.979	24.756	0.062
ISM1	0.315	0.830	242.542	<0.001	0.249	0.380	0.833

B score: B-score normalization; OR: odds ratio; F score: f-score normalization; CI: confidence interval; HDL: high-density lipoprotein, LDL: low-density lipoprotein, TG: triglycerides; BMI: body mass index, ISM1: isthmin-1;

*analysis of variance procedures within Statistical Package for the Social Sciences.

**Table 4.** TNM staging of the patient group.

Tumor (T) staging	n (%)	p-value
T1	10 (16.6)	0.718
T2	4 (6.6)	
T3	28 (46.8)	
T4	18 (30.0)	
Node (N) staging	n (%)	p-value
N 0	23 (38.3)	0.875
N1	6 (10.0)	
N2	13 (21.7)	
N3	18 (30.0)	
Metastasis (M) staging	n (%)	p-value
M0		
M1		

### Immunohistochemical findings

Light microscopy assessment of ISM1 immunoreactivity uncovered comparable staining patterns within the control (Figure 1) and intestinal metaplasia groups (Figure 2), with no statistically significant difference (p=0.998). However, noteworthy contrasts in ISM1 immunoreactivity were observed between the adenocarcinoma and the control and intestinal metaplasia groups (p<0.05).

**Figure 1.**
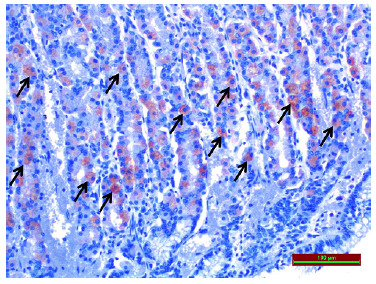
Isthmin-1 immunoreactivity in the control group.

Source: Hospital records.

**Figure 2.**
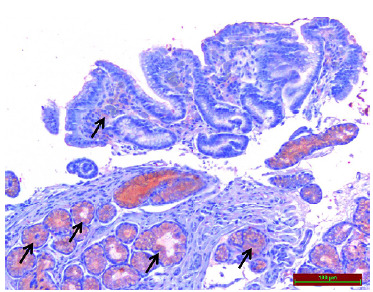
Isthmin-1 immunoreactivity in the intestinal metaplasia group.

Source: Hospital records.

**Figure 3.**
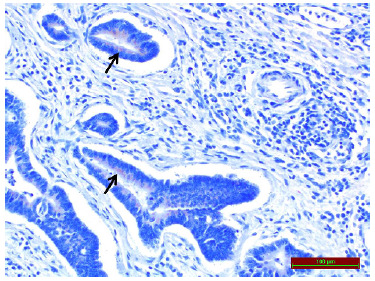
Isthmin-1 immunoreactivity in well-differentiated gastric adenocarcinoma.

Source: Hospital records.

**Figure 4.**
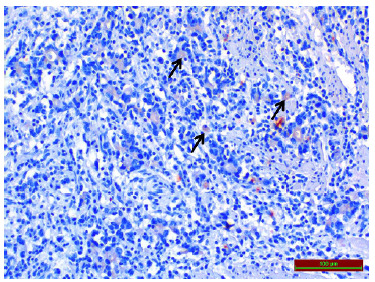
Isthmin-1 immunoreactivity in moderately-differentiated gastric adenocarcinoma.

Source: Hospital records.

**Figure 5.**
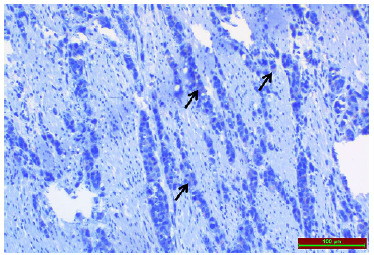
Isthmin-1 immunoreactivity in poorly-differentiated gastric adenocarcinoma.

Source: Hospital records.

Compared with the control group, significantly reduced ISM1 immunoreactivity was demonstrated in well-differentiated (Figure 3), moderately differentiated (Figure 4), and poorly differentiated (Figure 5) gastric adenocarcinoma subgroups (p<0.001 for all comparisons).

However, no significant differences in ISM1 expression were observed among the well-differentiated, moderately differentiated, and poorly differentiated adenocarcinoma subgroups (p*=*0.988) (Table 5).

**Table 5.** Isthmin-1 immunoreactivity and gastric adenocarcinoma differentiation.

Groups	ISM1 immunoreactivity histoscore	p-value
Control	1.480±0.534	
Intestinal metaplasia	1.445±0.538	
Adenocarcinoma		
Well-differentiated	0.322±0.096	<0.001*
Moderately-differentiated	0.302±0.099	
Poorly-differentiated	0.310±0.109	

ISM1: isthmin-1.

*p<0.05 compared with the control group (ANOVA test).

## DISCUSSION

Gastric cancer arises from a multifaceted interplay between environmental exposures and genetic factors. Despite global declines in incidence, it remains one of the most lethal malignancies worldwide^14^. Well-known predisposing conditions include *Helicobacter pylori* infection, increased age, high salt consumption, and diets deficient in fruits and vegetables. Although modern diagnostic tools such as endoscopy have contributed to earlier detection, the disease is still frequently identified at advanced stages due to its asymptomatic or nonspecific clinical presentation. The diagnostic process generally begins with histopathological evaluation following endoscopic biopsy, while staging is completed using imaging techniques including computed tomography, endoscopic ultrasound, positron emission tomography (PET), and laparoscopy^17^.

Although notable advancements have been made in nonsurgical treatments such as radiotherapy, chemotherapy, and immunotherapy, surgical resection remains the cornerstone of curative treatment for gastric cancer^5^.

Adipokines are regulatory peptides produced by adipose tissue, which act either locally or systemically to influence various biological processes. They are involved in metabolic regulation, immune system activity, inflammatory responses, vascular function, and redox balance^1^ ^,^ ^11^.

ISM1, a recently identified adipokine, has demonstrated tumor-suppressive functions in various models. Xiang et al. observed that ISM1 expression in melanoma cells reduced tumor vascularization and growth in mice^20^. ISM1, secreted by mature brown and white adipocytes, also plays a role in regulating glucose and lipid metabolism and has been implicated in metabolic diseases such as type 2 diabetes and hepatic steatosis^11^. In mouse models, ISM1 overexpression has been shown to inhibit lipid accumulation in the liver while enhancing the uptake of glucose and lipids, suggesting its role in maintaining metabolic balance^11^.

Holmgaard et al. identified ISM1 as a naturally occurring inhibitor of angiogenesis, relevant in both physiological and pathological conditions^10^. Furthermore, Osório et al. demonstrated that ISM1 can induce endothelial cell apoptosis and suppress tumor progression and vascularization when overexpressed in melanoma models^16^. These findings imply that elevated ISM1 expression may be part of the body’s defense mechanism against cancer, while reduced ISM1 activity could create a permissive environment for tumorigenesis.

Additional studies, including one by Lee et al., indicate that ISM1 can impair mitochondrial adenosine triphosphate production, triggering apoptotic pathways as part of its antitumor action^13^. Similarly, Chen et al. revealed that ISM1 activates two apoptosis pathways via surface receptors GRP78 and αvß5 integrin^4^. Contrary to these findings, Wu et al. reported an upregulation of ISM1 in colorectal cancer tissues based on ribonucleic acid analysis^19^. In the present study ISM1 levels were found to be significantly lower in gastric cancer tissues compared to healthy control samples, as determined through immunohistochemical analysis.

Notably, no statistically significant variation in ISM1 expression was found between different histological grades of gastric adenocarcinoma, including well-, moderately-, and poorly-differentiated subtypes.

## CONCLUSIONS

No significant difference in ISM1 levels was found between the control group and the premalignant cases. However, when adenocarcinoma cases were compared with the control and intestinal metaplasia groups, a significant decrease in ISM1 immunoreactivity was observed in adenocarcinoma cases. No significant difference was found between the staging and differentiation of adenocarcinoma cases, which were classified into three groups: well-differentiated, moderately differentiated, and poorly differentiated according to TNM staging. Based on these results, it is suggested that ISM1 could be used as a biomarker in the diagnosis of gastric carcinoma in the future. However, further comprehensive studies are needed to determine its usefulness in tumor staging or prognostic monitoring.

## DATA AVAILABILITY

The datasets generated and/or analyzed during the current study are available from the corresponding author upon reasonable request.
